# Antibacterial Activity of Silver and Its Application in Dentistry, Cardiology and Dermatology

**DOI:** 10.3390/microorganisms8091400

**Published:** 2020-09-11

**Authors:** Jasminka Talapko, Tatjana Matijević, Martina Juzbašić, Arlen Antolović-Požgain, Ivana Škrlec

**Affiliations:** 1 Faculty of Dental Medicine and Health, Josip Juraj Strossmayer University of Osijek, HR-31000 Osijek, Croatia; jtalapko@fdmz.hr (J.T.); martina.juzbasic@fdmz.hr (M.J.); 2 Department of Dermatology and Venereology, Clinical Hospital Center Osijek, HR-31000 Osijek, Croatia; tatjana.7.kovacevic@gmail.com; 3 Faculty of Medicine, Josip Juraj Strossmayer University of Osijek, HR-31000 Osijek, Croatia; 4 Department of Microbiology, Institute of Public Health Osijek, HR-31000 Osijek, Croatia

**Keywords:** silver nanoparticles, antibacterial activity, dentistry, cardiology, dermatology

## Abstract

The problem of antimicrobial resistance is increasingly present and requires the discovery of new antimicrobial agents. Although the healing features of silver have been recognized since ancient times, silver has not been used due to newly discovered antibiotics. Thanks to technology development, a significant step forward has been made in silver nanoparticles research. Nowadays, silver nanoparticles are a frequent target of researchers to find new and better drugs. Namely, there is a need for silver nanoparticles as alternative antibacterial nanobiotics. Silver nanoparticles (AgNPs), depending on their size and shape, also have different antimicrobial activity. In addition to their apparent antibacterial activity, AgNPs can serve as drug delivery systems and have anti-thrombogenic, anti-platelet, and anti-hypertensive properties. Today they are increasingly used in clinical medicine and dental medicine. This paper presents silver antimicrobial activity and its use in dentistry, cardiology, and dermatology, where it has an extensive range of effects.

## 1. Introduction

The issue of antimicrobial resistance is at the top of today’s problems [1] and is most often the result of the irrational use of antibiotics [2]. Solving this problem should be approached in a multidisciplinary way, including finding new agents with antimicrobial activity [3]. Silver, although its antibacterial activity has been recognized since early times, is again the target of research by many scientists, and trends in the clinical use of silver are rising [4]. The most popular medical forms of silver used in modern medicine are silver nitrate, silver sulfadiazine, and colloidal silver [4].

The use of products with silver nanoparticles is continuously growing, thus improving the knowledge of the biological interactions of silver nanoparticles and its side effects [5]. Silver nanoparticles have been one of the most popular topics of study in recent decades because they have outstanding antimicrobial activity even at low concentrations [6]. Materials with silver nanoparticles are a therapeutic choice due to the rising prevalence of bacterial resistance to antimicrobial agents. All of this has inspired researchers to study how silver’s antimicrobial features could be improved and applied in antimicrobial therapy [4].

The present paper presents the antimicrobial activity of silver and silver nanoparticles (AgNPs) and their application in dental medicine, cardiology, and dermatology. Possible side effects after frequent and excessive exposure to AgNPs were also discussed.

## 2. History of the Use of Silver

The history of the use of silver dates back to ancient times [7]. First, it was used as jewelry, for body piercings, currency and food handling. The earliest medical use of silver was for water storage to prevent spoilage and keep it fresh for drink. It is known that ancient Greeks and Romans, as other ancient empires, used silver for this purpose [8]. The earliest therapeutic use of silver was recorded in 1500 BC in the Han dynasty in China [4]. Silver nitrate is described as a medicine in the Pharmacopoeia published in Rome in 69 BC, probably to prevent and treat wound infections [9,10]. After the realization that microbes cause infections that came in the 1800s, the medical use of silver as an antimicrobial agent was confirmed. It has been used in the treatment of burn injuries for over 200 years [8]. In addition, silver nitrate was used for an eye infection, and it was one of the three antibiotics choices in the USA in the 1970s [8].

However, the discovery of penicillin in 1928 by Alexander Fleming, started the era of modern antimicrobial therapy [11], and until the discovery of penicillin, silver was commonly used as an antimicrobial agent [7].

Although, various topical antimicrobials are available today, silver sulfadiazine and silver nitrate continue to have their place in burn treatment and modern silver dressings have a major role in modern wound healing since the late 20th century [8].

## 3. Silver Nanoparticles

Nanotechnology is a quickly progressing area with various uses in the biomedical sciences [6] and involves engineering and production of materials at the atomic and molecular level. Mainly, it refers to structures of 1–100 nm in size [6,12], which contain from 20 to 15,000 silver atoms [6].

Different methods of silver nanoparticles (AgNPs) synthesis produce AgNPs of variable size, shape, morphology, and even stability. There are three methods of AgNPs synthesis: a physical, chemical, and biological synthesis [5]. The most widely used method is the chemical reduction of silver salts with sodium citrate or sodium borohydrate [13].

AgNPs are widely used in biomedical applications due to their antibacterial, antifungal, antiviral, and anti-inflammatory effects [5]. The application of AgNPs is extensive, from food packaging, materials, food additives, textiles, electronics, household appliances, cosmetics, medical devices, water disinfectants, and sprays, all because of their antibacterial properties [14].

Although both AgNPs and silver ions have antimicrobial activity, the advantages of AgNPs over silver ions are that AgNPs are less toxic and have increased antimicrobial activity, because due to their size, AgNPs penetrate the bacterial cell more easily [4].

## 4. Antimicrobial Activity of Silver Nanoparticles

The main applications of silver nanoparticles (AgNPs) in medicine include their use in the diagnostic procedures, but the most significant use is in therapy due to their antimicrobial action [15].

Bactericidal and inhibitory silver activity on pathogenic microorganisms has been proven in many scientific studies [16]. Silver has an advantage over the majority of other antimicrobials because it has an enormous scope of activity. It is effective against various types of microorganisms: bacteria, viruses, fungi [17].

Conventional antibiotics have a bactericidal effect on average on six pathogens. AgNPs could have a bactericidal impact on 650 pathogens without promoting the mechanism of the resistance [18].

Antibacterial activity is more evident in Gram-negative bacteria than in Gram-positive bacteria. The specific structure of Gram-positive bacteria is a cell wall, which is much thicker, denser, and is built of a thick layer of peptidoglycan. Peptidoglycans have a negative charge, which can slow down silver nanoparticles’ actions and make bacteria relatively more resistant to silver [19].

Under in vitro conditions, the surface charge has shown to play a significant role in the bactericidal response of AgNPs against Gram-negative and Gram-positive bacteria. Amongst the various silver nanoparticles tested, those with a positive charge had the most potent antimicrobial activity against all tested bacterial species [20]. It is crucial to remark that numerous researchers have proven the synergistic and additive silver effects of conventional antibiotics [21]. Therefore, it requires a lower dose of antibiotics used in therapy, which reduces the toxic effects of the antibiotic itself [22].

The bactericidal activity, stability, and biocompatibility of silver nanoparticles depend on their size. Silver nanoparticles should not be larger than 50 nm, and those of 10 and 15 nm have increased activity [16]. Smaller AgNPs have a higher surface-to-nanoparticle volume proportion, which permits them to associate more with cell membranes than larger nanoparticles. The highest antimicrobial activity has silver nanoparticles in the range of 1 to 10 nm [23,24].

### 4.1. Mechanism of Antibacterial Activity of Silver Nanoparticles

There are three mechanisms by which silver acts on microorganisms. One mechanism is that silver cations penetrate the cell wall of bacteria and react with peptidoglycans [4].

Oxidative stress, which results from the binding of AgNPs to a bacterial cell causing the ions’ release, is the second form of antibacterial action of silver nanoparticles [25,26]. Silver nanoparticles can bind to membrane proteins, which can significantly affect membrane permeability. This may result in leakage of cell contents, i.e., uncontrolled transport across the cytoplasmic membrane [25]. AgNPs that bind to membrane proteins can affect the uptake and release of phosphate ions and thus disrupt the respiratory chain and energy production [25].

Inhibition of transcription occurs due to the penetration of AgNPs into the cell where they could associate with intracellular elements such as lipids, proteins, and DNA [25], i.e., they damage DNA and act on protein synthesis [15]. Reactive oxygen species can be a significant factor in cell membrane disruption and DNA modification. AgNPs continuously release silver ions, which is estimated to be the mechanism of destroying microorganisms [6]. Thus, the bactericidal activity of AgNPs is a consequence of their action on the bacterial cell, resulting in cell death [15].

### 4.2. Biofilm

Biofilm has been associated with many bacterial infections [27]. As stated by the National Institutes of Health, biofilm is present in about 65% of microbial infections and 80% of chronic infections [28]. In a biofilm, bacteria communicate with each other. It is characterized by the persistence of infection and resistance to antimicrobial drugs [29].

Bacteria in its sessile form are 100 to 1000 times less sensitive to antibiotics than planktonic cells. This is because of the impermeability of the EPS (extracellular polymeric substance) matrix for antibiotics. The result is a severe danger to public health [28].

Finding an innovative way of therapy is considered to be the greatest challenge of the 21st century [30]. The anti-biofilm activity of silver nanoparticles has been proven in numerous analyses [31]. The fact that 25.2 ± 4 nm silver nanoparticles effectively prevent *Pseudomonas aeruginosa* biofilm formation and act bactericidal in already formed biofilm structures implies that they could be used to prevent and treat biofilm-related infections [32].

## 5. Clinical Application of Silver

Progress in nanotechnology has enabled the application of silver nanoparticles, which has created new therapeutic possibilities and a potentially wide range of applications [33]. The use of AgNPs varies including cardiovascular implants, catheters, dental composites, therapeutic drugs, and many others [24,34,35,36,37].

### 5.1. Application of Silver Nanoparticles in Dental Medicine

Silver has been used in dentistry for over a century and is a crucial component in dental amalgam fillings [4]. It is used in reconstructive dentistry, as well as in implantology and the production of dentures. Biofilms on the surface of a dental implant can cause inflammatory lesions on the peri-implant mucosa, consequently increasing the risk of implant failure [13]. The main goal of using silver nanoparticles is to prevent infection during and after dental surgery, i.e., thanks to their antibacterial activity, microbial colonization through embedded biomaterials are reduced [4,38,39].

The antimicrobial features of silver nanoparticles have also been studied in detail in dental medicine. Based on the results of the research, there is a growing interest in AgNPs [40]. The oral cavity is an active ecosystem that is regularly colonized by diverse pathogenic microorganisms, so dental materials and implants have an increased risk of infection [13]. In vitro examinations show the unique antimicrobial silver nanoparticles’ action when bound to dental materials such as nanocomposites, acrylic resins, composite resins, adhesives, intracanal drugs and implant coatings [40]. They are also used to make membranes for guided tissue regeneration in periodontal treatment [6]. Smaller silver nanoparticles have increased antibacterial activity against oral anaerobic pathogenic bacteria [6]. It is important to note that AgNPs, thanks to their antitumor properties, have shown positive results in the treatment of oral cancer [40].

The application of silver preparations as a microbicide to stop dentin caries is becoming more common. In vitro experiments demonstrated the microbicidal effectiveness of silver diamine fluoride (SDF) on cariogenic microbes in a human dentin model. Besides, silver nanoparticles have also been recognized in in vitro studies to have a microbicidal impact against growth, adhesion, and biofilm development of *Streptococcus mutans* in human dentin models. SDF has an intense antimicrobial effect on dental plaque. It reduces the metabolism of carbohydrates in dental plaque and stimulates a different balance of plaque flora [41,42].

SDF has a bactericidal effect on cariogenic bacteria, largely *S. mutans*, inhibiting the increase in cariogenic biofilms on teeth. Additionally, SDF stimulates remineralization of demineralized enamel or dentin and inhibits collagenases (matrix metalloproteinases and cysteine cathepsins) and thus protects the collagen in dentin from demolition [43].

AgNPs, in combination with antibiotics, enhances bactericidal features. When inactive antibiotics are combined with AgNPs, they gain strong antibacterial activity against multidrug-resistant strains of bacteria [6].

Silver nanoparticles have a better bacteriostatic and bactericidal outcome, with five times lower concentration than chlorhexidine. When AgNPs are used in the proper concentration, it is a safe option than other chemically derived antimicrobials [44]. Dental materials with AgNPs are biocompatible and have no meaningful toxic or mutagenic consequences [6].

### 5.2. Application of Silver Nanoparticles in Cardiology

Cardiovascular diseases (CVD) are the most common cause of human death worldwide. The majority of the CVD-related deaths are due to atherosclerotic plaque occlusion [45]. The new approach for the diagnosis, prevention, and treatment of atherosclerosis involve nanoparticles [46]. The most prominent nanoproduct are silver nanoparticles [35].

Biomaterials play an important role in cardiology. Thus, a coronary artery stent significantly enhanced the treatment of heart attack by providing mechanical support to narrowed vessels [47]. Silver in cardiology was first used in a silicone heart valve coated with silver to dodge bacterial infection and decrease inflammation response [5,13,34,36,48]. Clinical trials showed that a heart valve coated with silver causes side effects. Silver induces hypersensitivity, represses regular fibroblast activity, and causes paravalvular outflow [34,36,48]. New nanocomposites with AgNPs and carbon for stents and heart valves have anti-thrombogenic and antibacterial characteristics [34,36,49,50]. The use of cardiac pacemakers, whose surfaces have been treated with AgNPs, dodges the application of antibiotic-loaded pouches. This approach decreases the risk of infection in the first few months after surgery and increases the likelihood of a positive outcome for patients [51]. The study of de Mel et al. showed that polymeric materials for cardiovascular implants with integrated AgNPs have antibacterial and anti-thrombogenic properties [52]. The potential application of AgNPs in cardiology is to use them as a vehicle to deliver drugs at a specific site in the organism [46,53].

Silver ions, generally in the form of a silver salt, show much higher toxicity than any size of AgNPs [14]. In the cardiovascular system, Ag ionic form, such as silver chloride and silver nitrate, caused cardiac changes in rats such as left ventricular hypertrophy [54], causing hypersensitivity and inhibiting regular fibroblast function and causing paravalvular outflow in patients [5,55]. AgNPs are secure and harmless in biomedical implants, as opposed to silver ions. [5]. Although AgNPs are also partially soluble and release Ag ions [56]. Moreover, cationic coatings (positive charge) for AgNPs cause higher toxicity than anionic and neutral coatings [14]. AgNPs could be detected in the cardiovascular system after inhalation, dermal exposure, oral exposure, or direct injection. Inhaled AgNPs, which penetrate in circulation, are associated with cardiovascular functions such as cardiac rhythm disorders and coagulation [35,54,57,58].

The effect of AgNPs depend on their size, dose, and exposure time [54]. Data on AgNPs’ effects are debatable because they described potential toxicity and possible advantages [54]. Some studies showed that AgNPs have antiplatelet properties [47,59,60]. AgNPs stimulates the generation of vascular endothelial growth factor (VEGF), which is connected with the production of new blood vessels or angiogenesis [13,54]. Another potential of AgNPs is the induction of endothelial vasodilatation and therefore improved blood flow in the heart [13,54]. So it could be used as a potential antihypertensive agent [54]. The nanocomposite with AgNPs and multilayer films containing AgNPs have antibacterial, mechanical, and hemodynamic properties in cardiovascular implant coating [5,49,50,61].

The toxicity of AgNPs is not only connected with the release of silver from the nanomaterials [62]. Both oxidative stress and production of reactive oxygen species (ROS) have central role in AgNPs toxicity [46,54,63,64,65,66,67,68]. AgNPs damage cellular components and lead to DNA damage, and damage to the cell membrane [36,69], which are the hallmarks of early apoptosis. AgNPs increase the ROS production, which is responsible for myocardial damage [63,64,67], and simultaneously reduces the production of nitric oxide [67]. Silver nanoparticles promoted oxidative stress and DNA destruction in human endothelial cells. Those data suggest that the AgNPs were toxic to endothelial precursors that participate in angiogenesis [70,71]. ROS could harm the heart muscle and lead to inflammation and oxidative stress in rats [63,72,73].

AgNPs induced erythrocytes hemolysis, platelet aggregation, and procoagulant activation [71]. Exposure to AgNPs could lead to primary atherosclerosis [14,74].

Exposure to AgNPs might cause damage to the heart, bone, and kidney. The raised dermal application of AgNPs induced deformation of the cardiocytes and inflammation [73]. AgNPs are hazardous to cardiac electro-physiology and can lead to lethal bradyarrhythmias and cardiac arrest [75]. Cardiovascular disorders, such as hypertension, may affect the harmful consequences caused by AgNPs [13,67].

Studies on animals revealed that inhaled AgNPs are disseminated to all body organs [63,76]. In the rats, AgNPs increased heart rate and reduced dilation of the artery [71,77]. Dependent on the dose, AgNPs can induce vasodilation or vasoconstriction in separated rat aortic rings [71,78]. In rats, AgNPs could pass the blood–brain barrier inducing necrosis and neuronal degeneration [36,71,79]. AgNPs affect the beginning of fish embryos development. It causes chromosomal aberrations and DNA damage, and prevent the generation of zebrafish. Those data show that AgNPs might have possible teratogenic consequences in human [36,80]. In some endothelial cells, AgNPs activates apoptosis through increased caspase three activity [54].

Similar to all drugs and medical devices, a crucial evaluation of the advantages and disadvantages of AgNPs is needed to ensure their safety as a therapeutic agent.

### 5.3. Application of Silver Nanoparticles in Dermatology

Thanks to its antimicrobial properties, silver has well-established use in dermatology. It is an active antimicrobial agent with broad-spectrum activity, so it is used to prevent and treat infections in acute wounds (such as traumatic, surgical, and burn injuries) and chronic wounds (such as diabetic foot ulcers, pressure ulcers, venous leg ulcers) [81]. When in contact with wound fluid, metallic silver salt (Ag^0^) becomes ionized (Ag^+^) and highly active against bacteria. Because only the ionized form of silver has desired antiseptic properties, contact with wound fluid is necessary if the source is metallic silver [82]. Silver has been incorporated into various dressing products in addition to creams, gels, and barrier protectants, which differ in their solubility and the rate at which silver ions are released into the wound bed. Nanocrystalline silver dressings were launched commercially as antimicrobial dressings in 1998 [83]. They are designed for sustained silver release, releasing antibacterial silver levels for 3–7 days, resulting in less frequent re-application of silver preparations or dressings [84]. Another benefit of silver use in modern wound management is the availability of various products for different wound situations (e.g., gauzes, hydrocolloids, hydrogels, alginates, foams). Wound characteristics which determine the choice of suitable dressing are: depth, amount of exudate, bleeding, pain. Providing a moist wound environment is an essential principle of wound healing. In addition, silver dressings appear to decrease matrix metalloproteinases that are upregulated in non-healing, chronic wounds [85]. They may also promote cellular proliferation and re-epithelialization by inducing the production of metallothionein by epidermal cells. Metallothionein increases zinc- and copper-dependent enzymes required for cellular proliferation and matrix remodeling [82].

Because of numbered qualities, the use of silver dressings can reduce the treatment time and thus lead to cost savings compared to the treatment of silver-free dressings. However, published reviews found different results regarding their effectiveness. Silver-containing dressings improve the likelihood of healing venous leg ulcers, as confirmed by the 2018 Cochrane review [86]. On the other hand, many studies found no significantly higher healing rates [87,88]. Considering all that and their relatively high price, in modern wound management, the use of silver dressings is supported only if there are symptoms of wound infection.

Topical silver preparation, silver sulfadiazine (SSD) as a 1% cream, applied once to twice a day, is usually used in partial-thickness and full-thickness burns [81]. It was discovered in the 1960s. Based on systematic reviews from 2014, 2017, and 2018, it was presumed that more advanced methods, with and without silver, lead to more improved wound healing and infection-prevention than silver sulfadiazine [89,90]. Therefore, SSD is no longer so often recommended as it used to be. There is also a risk of toxicity to host cells (fibroblasts and keratinocytes) [81]. Silver sulfadiazine 1% cream is sulfa-drug, a group of synthetic antibiotics containing the sulfonamide molecular structures. Allergic reactions to sulfa drugs are among the most common drug allergies, so they should be prescribed with caution. Other uncommon adverse effects of silver sulfadiazine are hemolysis in patients with glucose-6-dehydrogenase (G6PD) deficiency, hyperosmolality, methemoglobinemia, and leukopenia (neutropenia) in children [91]. These conditions are reversible once the cream is discontinued. Silver sulfadiazine has a low toxicity profile, but the application to large burn sites or prolonged use in bullous disorders should be avoided [92]. Argyria is a blue-purple-gray discoloration of the skin produced by silver deposition and can be localized or generalized. Argyria is not treatable or reversible. There are also a few reported cases of argyria secondary to silver dressings [93]. Topical incorporation of silver into the skin depends on the vehicle used, concentration, particle size and shape, substance type (depending if the source of silver is salt or nanoparticle). Smaller nanometer particles are better able to penetrate the skin than larger particles [94].

Another silver preparation that has widespread use in dermatology is silver nitrate (AgNO_3_). It is used for its caustic actions, in solid form or solutions, more durable than 5%. In clinical practice, it is often used to treat small recalcitrant ulcerations or to diminish excess granulation tissue, also called hypergranulation, which can negatively influence wound healing [81,95]. Silver nitrate is also used effectively to treat warts and *molluscum contagiosum*, which are common viral infections, especially among children [96]. In one study, 389 sequential patients with *molluscum contagiosum* were prescribed 40% silver nitrate paste with an excellent cure rate [97]. Silver nitrate is also used to stop bleeding in small superficial wounds after curettage or shaving lesions in dermatological surgery. Although it is used because of its astringent and caustic features, caution is needed because the depth of injury can be increased [96].

Silver also has anti-inflammatory effects and may have angiogenic properties. Its action on the cytokine system mediates the anti-inflammatory properties of silver [83]. Acne is a chronic inflammatory condition of the pilosebaceous units, and the Gram-positive *Propionibacterium acnes* bacterium is believed to have a crucial function in the pathophysiology [98]. Because of the combination of anti-inflammatory and antimicrobial activity, it was assumed that topical silver preparations would benefit acne vulgaris. A small number of studies were conducted to test this hypothesis [99]. Soaps with nanosilver are broadly applied in the medication of acne. In conclusion, silver preparations are often used “off-label” in this indication due to the low possibility of developing bacterial resistance, the absence of irritation, and the preservation of the skin barrier.

Atopic dermatitis (AD) is the most widespread chronic inflammatory skin disease marked by pruritus and relapsing course [100,101]. It is also known as eczema and atopic eczema. More than 90% of patients with atopic dermatitis have skin colonized with *Staphylococcus aureus*, compared to about 5% of the unaffected individuals [102]. Silver has an excellent antibacterial effect on *S. aureus*, so it is presumed to enhance AD’s clinical signs and symptoms. In a few studies, the use of silver-coated textiles in patients with AD was analyzed. Most of them demonstrated that approximately seven days of wearing such textiles could significantly diminish *S. aureus* density and improve AD symptoms compared with wearing cotton [103]. On the other side, it has its disadvantages. Washing of silver-infused textiles is one of them. The amount of silver lost from textiles can range from 100% loss after four washes to less than 1%. Additionally, there is a possibility that textile silver ends up in the water supply, reducing the number of beneficial bacteria used to treat it [104].

Because of the increasing resistance of fungal strains, including dermatophyte strains, there is an urgent need for novel antifungals. So, the antifungal activity of AgNPs has been tested. In one study, it was effective against *Trichophyton violaceum*, but not against *Microsporum canis* or *Microsporum gypseum* [105]. Mousavi et al. also found that *M. canis* was more resistant to silver nanoparticles [106]. Atef et al. reported the growing inhibition of the silver nanoparticles on *Trichophyton mentagrophytes* and *Candida albicans* [107]. Some researchers also compared the antifungal activity of AgNPs with the current antifungals. Mousavi et al. found that griseofulvin had higher anti-dermatophyte activity than silver nanoparticles [106]. Others showed that silver nanoparticles had superior efficiency compared with fluconazole and less antifungal efficiency than griseofulvin. However, they also showed that the antifungal outcomes of fluconazole and griseofulvin were enhanced in the presence of the silver nanoparticles [108]. In conclusion, the antifungal activity of AgNPs is yet to be confirmed with more similar studies.

## 6. Side Effects of Silver Nanoparticles

Silver nanoparticles have many advantages and numerous biomedical applications, but their side effects have been increasingly studied [13]. Different studies on the side effects of silver nanoparticles conducted in different biological systems have contradictory and different results [5,6,13,14,109]. AgNPs can stimulate the formation and intracellular accumulation of reactive oxygen species, leading to mitochondrial membrane permeability and DNA damage. Oxidative stress caused by silver nanoparticles could result in inflammatory responses [5,13,14]. AgNPs’ side effects are associated with free silver ions, although AgNPs could cross the blood–brain barrier [6] and have harmful effects on short- and long-term memory [14].

Inhalation of AgNPs accumulates in the blood, liver, lungs, kidneys, stomach, testes, and brain, but AgNPs do not have vital genotoxicity after oral administration of AgNPs [5]. Smaller silver nanoparticles have more significant side effects than larger ones due to larger surface area and reactivity [14]. AgNPs could undergo different alterations before they end up in the environment. Adverse biotoxic effects of silver are based on the type of silver compound present in the environment [14].

AgNPs are efficient in destroying microorganisms. However, it could cause the same damage to healthy cells and the ecosystem, mainly if not used under supervision and without a thorough risk assessment [110]. The clinical importance of the possible toxicity of AgNPs remains unexplained. Clinical evidence is contradictory, and further research is needed.

## 7. Conclusions

The antimicrobial activity of AgNPs is very pronounced, and it is particularly significant that they can overcome the usual mechanisms of resistance formation. Combined with conventional antibiotics, they have a synergistic and additive effect on bacteria, showing particular effectiveness against multidrug-resistant bacteria and are useful against biofilm. It is these two segments that represent the most significant problems of modern medicine.

The prevalence of silver nanoparticles in dentistry is increasing. It is used in implant treatment, as well as in orthodontic, endodontic, restorative, prosthetic, and periodontal treatment. Anti-caries action, as well as antitumor activity in the oral cavity, were also observed.

In dermatology, silver is used in modern wound management in the form of silver dressings that are effective and with notable regenerative potential. Different silver preparations are used in everyday practice due to its astringent and caustic features. Because of the mentioned qualities, silver-based products also have great potential in therapy for various skin conditions: acne, eczema, fungal infections, etc.

In cardiology, for cardiovascular implants, polymeric materials with integrated AgNPs are used because they have antibacterial and anti-thrombogenic properties. However, similar to all drugs and medical devices, a crucial assessment of the advantages and disadvantages of AgNPs is needed to ensure their safety as a therapeutic agent. The long-term effects of the use or exposure to AgNPs could be harmful. Therefore, AgNPs should only be used when there is an absolute need for it or when silver is immobilized, and there is no risk from toxic free silver ions.

Considering that AgNPs are cheap and have low cytotoxicity, they represent an alternative antimicrobial nanobiotic.

## References

[1] Prestinaci F., Pezzotti P., Pantosti A. (2015). Antimicrobial resistance: A global multifaceted phenomenon. Pathog. Glob. Health.

[2] Mboya E.A., Sanga L.A., Ngocho J.S. (2018). Irrational use of antibiotics in the moshi municipality Northern Tanzania: A cross sectional study. Pan Afr. Med. J..

[3] Talapko J., Drenjančević D., Stupnišek M., Tomić Paradžik M., Kotris I., Belić D., Bogdan M., Sikirić P. (2018). In vitro evaluation of the antibacterial activity of pentadecapeptide BPC 157. Acta Clin. Croat..

[4] Sim W., Barnard R.T., Blaskovich M.A.T., Ziora Z.M. (2018). Antimicrobial silver in medicinal and consumer applications: A patent review of the past decade (2007–2017). Antibiotics.

[5] Ge L., Li Q., Wang M., Ouyang J., Li X., Xing M.M.Q. (2014). Nanosilver particles in medical applications: Synthesis, performance, and toxicity. Int. J. Nanomed..

[6] Yin I.X., Zhang J., Zhao I.S., Mei M.L., Li Q., Chu C.H. (2020). The Antibacterial Mechanism of Silver Nanoparticles and Its Application in Dentistry. Int. J. Nanomed..

[7] Ahmed S., Ahmad M., Swami B.L., Ikram S. (2016). A review on plants extract mediated synthesis of silver nanoparticles for antimicrobial applications: A green expertise. J. Adv. Res..

[8] Barillo D.J., Marx D.E. (2014). Silver in medicine: A brief history BC 335 to present. Burns.

[9] Gao S.S., Zhao I.S., Duffin S., Duangthip D., Lo E.C.M., Chu C.H. (2018). Revitalising silver nitrate for caries management. Int. J. Environ. Res. Public Health.

[10] Calvery H.O., Lightbody H.D., Rones B. (1941). Effects of some silver salts on the eye: (Silver nitrate, silver ammonium nitrate, silver ammonium sulfate, silver ammonium lactate and a mixture of silver ammonium nitrate and silver ammonium sulfate). Arch. Ophthalmol..

[11] Talapko J., Škrlec I., Alebić T., Bekić S., Včev A. (2018). From Bacteriophage to Antibiotics and Back. Coll. Antropol..

[12] Farokhzad O.C., Langer R. (2009). Impact of nanotechnology on drug delivery. ACS Nano.

[13] Burdușel A.C., Gherasim O., Grumezescu A.M., Mogoantă L., Ficai A., Andronescu E. (2018). Biomedical applications of silver nanoparticles: An up-to-date overview. Nanomaterials.

[14] Rezvani E., Rafferty A., McGuinness C., Kennedy J. (2019). Adverse effects of nanosilver on human health and the environment. Acta Biomater..

[15] Mathur P., Jha S., Ramteke S., Jain N.K. (2018). Pharmaceutical aspects of silver nanoparticles. Artif. Cells Nanomed. Biotechnol..

[16] Dakal T.C., Kumar A., Majumdar R.S., Yadav V. (2016). Mechanistic basis of antimicrobial actions of silver nanoparticles. Front. Microbiol..

[17] Mcdonnell G., Russell A.D. (1999). Antiseptics and disinfectants: Activity, action, and resistance. Clin. Microbiol. Rev..

[18] Wang L., Hu C., Shao L. (2017). The antimicrobial activity of nanoparticles: Present situation and prospects for the future. Int. J. Nanomed..

[19] Domínguez A.V., Algaba R.A., Canturri A.M., Villodres Á.R., Smani Y. (2020). Antibacterial activity of colloidal silver against gram-negative and gram-positive bacteria. Antibiotics.

[20] Abbaszadegan A., Ghahramani Y., Gholami A., Hemmateenejad B., Dorostkar S., Nabavizadeh M., Sharghi H. (2015). The effect of charge at the surface of silver nanoparticles on antimicrobial activity against gram-positive and gram-negative bacteria: A preliminary study. J. Nanomater..

[21] Abo-Shama U.H., El-Gendy H., Mousa W.S., Hamouda R.A., Yousuf W.E., Hetta H.F., Abdeen E.E. (2020). Synergistic and antagonistic effects of metal nanoparticles in combination with antibiotics against some reference strains of pathogenic microorganisms. Infect. Drug Resist..

[22] Zou L., Wang J., Gao Y., Ren X., Rottenberg M.E., Lu J., Holmgren A. (2018). Synergistic antibacterial activity of silver with antibiotics correlating with the upregulation of the ROS production. Sci. Rep..

[23] Shang L., Nienhaus K., Nienhaus G.U. (2014). Engineered nanoparticles interacting with cells: Size matters. J. Nanobiotechnol..

[24] Škrlová K., Malachová K., Muñoz-Bonilla A., Měřinská D., Rybková Z., Fernández-García M., Plachá D. (2019). Biocompatible polymer materials with antimicrobial properties for preparation of stents. Nanomaterials.

[25] Tang S., Zheng J. (2018). Antibacterial Activity of Silver Nanoparticles: Structural Effects. Adv. Healthc. Mater..

[26] Möhler J.S., Sim W., Blaskovich M.A.T., Cooper M.A., Ziora Z.M. (2018). Silver bullets: A new lustre on an old antimicrobial agent. Biotechnol. Adv..

[27] Wu H., Moser C., Wang H.Z., Høiby N., Song Z.J. (2015). Strategies for combating bacterial biofilm infections. Int. J. Oral Sci..

[28] Medici S., Peana M., Nurchi V.M., Zoroddu M.A. (2019). Medical Uses of Silver: History, Myths, and Scientific Evidence. J. Med. Chem..

[29] Chen L., Wen Y.M. (2011). The role of bacterial biofilm in persistent infections and control strategies. Int. J. Oral Sci..

[30] Vraneš J., Leskovar V. (2009). Značenje nastanka mikrobnog biofilma u patogenezi i liječenju kroničnih infekcija. Med. Glas. Ljek. Komore Zenicko-Doboj. Kantona.

[31] Hamed A.A., Kabary H., Khedr M., Emam A.N. (2020). Antibiofilm, antimicrobial and cytotoxic activity of extracellular green-synthesized silver nanoparticles by two marine-derived actinomycete. RSC Adv..

[32] Markowska K., Grudniak A., Wolska K. (2013). Silver nanoparticles as an alternative strategy against bacterial biofilms. Acta Biochim. Pol..

[33] Wong K.K.Y., Liu X. (2010). Silver nanoparticles—The real “silver bullet” in clinical medicine?. Medchemcomm.

[34] Rafique M., Sadaf I., Rafique M.S., Tahir M.B. (2017). A review on green synthesis of silver nanoparticles and their applications. Artif. Cells Nanomed. Biotechnol..

[35] Chen X., Schluesener H.J. (2008). Nanosilver: A nanoproduct in medical application. Toxicol. Lett..

[36] Murphy M., Ting K., Zhang X., Soo C., Zheng Z. (2015). Current Development of Silver Nanoparticle Preparation, Investigation, and Application in the Field of Medicine. J. Nanomater..

[37] Song W.H., Hyun S.R., Hong S.H. (2009). Antibacterial properties of Ag (or Pt)-containing calcium phosphate coatings formed by micro-arc oxidation. J. Biomed. Mater. Res. Part A.

[38] Corrêa J.M., Mori M., Sanches H.L., Cruz A.D.D., Poiate E., Poiate I.A.V.P. (2015). Silver nanoparticles in dental biomaterials. Int. J. Biomater..

[39] Fakhruddin K.S., Egusa H., Ngo H.C., Panduwawala C., Pesee S., Samaranayake L.P. (2020). Clinical efficacy and the antimicrobial potential of silver formulations in arresting dental caries: A systematic review. BMC Oral Health.

[40] Noronha V.T., Paula A.J., Durán G., Galembeck A., Cogo-Müller K., Franz-Montan M., Durán N. (2017). Silver nanoparticles in dentistry. Dent. Mater..

[41] Liu B.Y., Liu J., Zhang D., Yang Z.L., Feng Y.P., Wang M. (2020). Effect of silver diammine fluoride on micro-ecology of plaque from extensive caries of deciduous teeth—In vitro study. BMC Oral Health.

[42] Lou Y., Darvell B., Botelho M. (2018). Antibacterial Effect of Silver Diamine Fluoride on Cariogenic Organisms. J. Contemp. Dent. Pract..

[43] Zhao I.S., Gao S.S., Hiraishi N., Burrow M.F., Duangthip D., Mei M.L., Lo E.C.M., Chu C.H. (2018). Mechanisms of silver diamine fluoride on arresting caries: A literature review. Int. Dent. J..

[44] Panpaliya N.P., Dahake P.T., Kale Y.J., Dadpe M.V., Kendre S.B., Siddiqi A.G., Maggavi U.R. (2019). In vitro evaluation of antimicrobial property of silver nanoparticles and chlorhexidine against five different oral pathogenic bacteria. Saudi Dent. J..

[45] World Health Organization (2018). Noncommunicable Diseases Country Profiles 2018.

[46] Bejarano J., Navarro-Marquez M., Morales-Zavala F., Morales J.O., Garcia-Carvajal I., Araya-Fuentes E., Flores Y., Verdejo H.E., Castro P.F., Lavandero S. (2018). Nanoparticles for diagnosis and therapy of atherosclerosis and myocardial infarction: Evolution toward prospective theranostic approaches. Theranostics.

[47] Jiang W., Rutherford D., Vuong T., Liu H. (2017). Nanomaterials for treating cardiovascular diseases: A review. Bioact. Mater..

[48] Grunkemeier G.L., Jin R., Starr A. (2006). Prosthetic Heart Valves: Objective Performance Criteria Versus Randomized Clinical Trial. Ann. Thorac. Surg..

[49] Andara M., Agarwal A., Scholvin D., Gerhardt R.A., Doraiswamy A., Jin C., Narayan R.J., Shih C.C., Shih C.M., Lin S.J. (2006). Hemocompatibility of diamondlike carbon-metal composite thin films. Diam. Relat. Mater..

[50] Ghanbari H., Viatge H., Kidane A.G., Burriesci G., Tavakoli M., Seifalian A.M. (2009). Polymeric heart valves: New materials, emerging hopes. Trends Biotechnol..

[51] Shawcross J., Bakhai A., Ansaripour A., Armstrong J., Lewis D., Agg P., De Godoy R., Blunn G. (2017). In vivo biocompatibility and pacing function study of silver ion-based antimicrobial surface technology applied to cardiac pacemakers. Open Heart.

[52] De Mel A., Chaloupka K., Malam Y., Darbyshire A., Cousins B., Seifalian A.M. (2012). A silver nanocomposite biomaterial for blood-contacting implants. J. Biomed. Mater. Res. Part A.

[53] Bamrungsap S., Zhao Z., Chen T., Wang L., Li C., Fu T., Tan W. (2012). Nanotechnology in therapeutics: A focus on nanoparticles as a drug delivery system. Nanomedicine.

[54] Gonzalez C., Rosas-Hernandez H., Ramirez-Lee M.A., Salazar-García S., Ali S.F. (2014). Role of silver nanoparticles (AgNPs) on the cardiovascular system. Arch. Toxicol..

[55] Jamieson W.R.E., Fradet G.J., Abel J.G., Janusz M.T., Lichtenstein S.V., MacNab J.S., Stanford E.A., Chan F. (2009). Seven-year results with the St Jude Medical Silzone mechanical prosthesis. J. Thorac. Cardiovasc. Surg..

[56] Cao Y., Gong Y., Liao W., Luo Y., Wu C., Wang M., Yang Q. (2018). A review of cardiovascular toxicity of TiO2, ZnO and Ag nanoparticles (NPs). BioMetals.

[57] Peters K., Unger R.E., Kirkpatrick C.J., Gatti A.M., Monari E. (2004). Effects of nano-scaled particles on endothelial cell function in vitro: Studies on viability, proliferation and inflammation. J. Mater. Sci. Mater. Med..

[58] Karg E.W., Takenaka S., Karg E., Roth C., Schulz H., Ziesenis A., Heinzmann U., Schramel P., Heyder J. (2001). Pulmonary and Systemic Distribution of Inhaled Ultrafine Silver Particles in Rats Pulmonary and Systemic Distribution of Inhaled Ultrafine Silver Particles in Rats. Environ. Health Perspect..

[59] Shrivastava S., Bera T., Singh S.K., Singh G., Ramachandrarao P., Dash D. (2009). Characterization of antiplatelet properties of silver nanoparticles. ACS Nano.

[60] Bandyopadhyay D., Baruah H., Gupta B., Sharma S. (2012). Silver nano particles prevent platelet adhesion on immobilized fibrinogen. Indian J. Clin. Biochem..

[61] Fu J., Ji A., Fan D., Shen J. (2006). Construction of antibacterial multilayer films containing nanosilver via layer-by-layer assembly of heparin and chitosan-silver ions complex. J. Biomed. Mater. Res. Part A.

[62] Holland N., Becak D., Shannahan J., Brown J., Carratt S., Van Winkle L., Pinkerton K., Wang C., Munusamy P., Baer D. (2015). Cardiac Ischemia Reperfusion Injury Following Instillation of 20 nm Citrate-capped Nanosilver. J. Nanomed. Nanotechnol..

[63] Bostan H.B., Rezaee R., Valokala M.G., Tsarouhas K., Golokhvast K., Tsatsakis A.M., Karimi G. (2016). Cardiotoxicity of nano-particles. Life Sci..

[64] Zhou T., Chuang C.-C., Zuo L. (2015). Molecular Characterization of Reactive Oxygen Species in Myocardial Ischemia-Reperfusion Injury. Biomed Res. Int..

[65] Teodoro J.S., Silva R., Varela A.T., Duarte F.V., Rolo A.P., Hussain S., Palmeira C.M. (2016). Low-dose, subchronic exposure to silver nanoparticles causes mitochondrial alterations in Sprague-Dawley rats. Nanomedicine.

[66] Recordati C., De Maglie M., Bianchessi S., Argentiere S., Cella C., Mattiello S., Cubadda F., Aureli F., D’Amato M., Raggi A. (2016). Tissue distribution and acute toxicity of silver after single intravenous administration in mice: Nano-specific and size-dependent effects. Part. Fibre Toxicol..

[67] Ramirez-Lee M.A., Aguirre-Bañuelos P., Martinez-Cuevas P.P., Espinosa-Tanguma R., Chi-Ahumada E., Martinez-Castañon G.A., Gonzalez C. (2018). Evaluation of cardiovascular responses to silver nanoparticles (AgNPs) in spontaneously hypertensive rats. Nanomed. Nanotechnol. Biol. Med..

[68] Wang M., Yang Q., Long J., Ding Y., Zou X., Liao G., Cao Y. (2018). A comparative study of toxicity of TiO2, ZnO, and Ag nanoparticles to human aortic smooth-muscle cells. Int. J. Nanomed..

[69] McShan D., Ray P.C., Yu H. (2014). Molecular toxicity mechanism of nanosilver. J. Food Drug Anal..

[70] Castiglioni S. (2014). Short- and long-term effects of silver nanoparticles on human microvascular endothelial cells. World J. Biol. Chem..

[71] Yu X., Hong F., Zhang Y.Q. (2016). Bio-effect of nanoparticles in the cardiovascular system. J. Biomed. Mater. Res. Part A.

[72] Ebabe Elle R., Gaillet S., Vidé J., Romain C., Lauret C., Rugani N., Cristol J.P., Rouanet J.M. (2013). Dietary exposure to silver nanoparticles in Sprague-Dawley rats: Effects on oxidative stress and inflammation. Food Chem. Toxicol..

[73] Korani M., Rezayat S.M., Bidgoli S.A. (2013). Sub-chronic Dermal Toxicity of Silver Nanoparticles in Guinea Pig: Special Emphasis to Heart, Bone and Kidney Toxicities. Iran. J. Pharm. Res..

[74] Shi J., Sun X., Lin Y., Zou X., Li Z., Liao Y., Du M., Zhang H. (2014). Endothelial cell injury and dysfunction induced by silver nanoparticles through oxidative stress via IKK/NF-κB pathways. Biomaterials.

[75] Lin C.X., Yang S.Y., Gu J.L., Meng J., Xu H.Y., Cao J.M. (2017). The acute toxic effects of silver nanoparticles on myocardial transmembrane potential, INa and IK1 channels and heart rhythm in mice. Nanotoxicology.

[76] Hadrup N., Lam H.R. (2014). Oral toxicity of silver ions, silver nanoparticles and colloidal silver—A review. Regul. Toxicol. Pharmacol..

[77] Roberts J.R., McKinney W., Kan H., Krajnak K., Frazer D.G., Thomas T.A., Waugh S., Kenyon A., MacCuspie R.I., Hackley V.A. (2013). Pulmonary and cardiovascular responses of rats to inhalation of silver nanoparticles. J. Toxicol. Environ. Health-Part A Curr. Issues.

[78] Rosas-Hernández H., Jiménez-Badillo S., Martínez-Cuevas P.P., Gracia-Espino E., Terrones H., Terrones M., Hussain S.M., Ali S.F., González C. (2009). Effects of 45-nm silver nanoparticles on coronary endothelial cells and isolated rat aortic rings. Toxicol. Lett..

[79] Tang J., Xiong L., Wang S., Wang J., Liu L., Li J., Wan Z., Xi T. (2008). Influence of silver nanoparticles on neurons and blood-brain barrier via subcutaneous injection in rats. Appl. Surf. Sci..

[80] Lee K.J., Nallathamby P.D., Browning L.M., Osgood C.J., Nancy Xu X.H. (2007). In vivo imaging of transport and biocompatibility of single silver nanoparticles in early development of zebrafish embryos. ACS Nano.

[81] Lipsky B.A., Hoey C. (2009). Topical Antimicrobial Therapy for Treating Chronic Wounds. Clin. Pract..

[82] Lansdown A.B.G. (2004). A review of the use of silver in wound care: Facts and fallacies. Br. J. Nurs..

[83] Nadworny P.L., Wang J., Tredget E.E., Burrell R.E. (2010). Anti-inflammatory activity of nanocrystalline silver-derived solutions in porcine contact dermatitis. J. Inflamm..

[84] Carter M.J., Tingley-Kelley K., Warriner R.A. (2010). Silver treatments and silver-impregnated dressings for the healing of leg wounds and ulcers: A systematic review and meta-analysis. J. Am. Acad. Dermatol..

[85] Widgerow A.D. (2010). Nanocrystalline silver, gelatinases and the clinical implications. Burns.

[86] Norman G., Westby M.J., Rithalia A.D., Stubbs N., Soares M.O., Dumville J.C. (2018). Dressings and topical agents for treating venous leg ulcers. Cochrane Database Syst. Rev..

[87] Vermeulen H., Van Hattem J.M., Storm-Versloot M.N., Ubbink D.T. (2007). Topical silver for treating infected wounds. Cochrane Database Syst. Rev..

[88] O’Meara S., Al-Kurdi D., Ologun Y., Ovington L.G., Martyn-St James M., Richardson R. (2014). Antibiotics and antiseptics for venous leg ulcers. Cochrane Database Syst. Rev..

[89] Rashaan Z.M., Krijnen P., Klamer R.R.M., Schipper I.B., Dekkers O.M., Breederveld R.S. (2014). Nonsilver treatment vs. silver sulfadiazine in treatment of partial-thickness burn wounds in children: A systematic review and meta-analysis. Wound Repair Regen..

[90] Nímia H.H., Carvalho V.F., Isaac C., Souza F.Á., Gemperli R., Paggiaro A.O. (2019). Comparative study of Silver Sulfadiazine with other materials for healing and infection prevention in burns: A systematic review and meta-analysis. Burns.

[91] Hadrup N., Sharma A.K., Loeschner K. (2018). Toxicity of silver ions, metallic silver, and silver nanoparticle materials after in vivo dermal and mucosal surface exposure: A review. Regul. Toxicol. Pharmacol..

[92] Mintz E., George D., Hsu S. (2008). Silver sulfadiazine therapy in widespread bullous disorders: Potential for toxicity. Dermatol Online J..

[93] De Lima R., Seabra A.B., Durán N. (2012). Silver nanoparticles: A brief review of cytotoxicity and genotoxicity of chemically and biogenically synthesized nanoparticles. J. Appl. Toxicol..

[94] Labouta H.I., Schneider M. (2013). Interaction of inorganic nanoparticles with the skin barrier: Current status and critical review. Nanomed. Nanotechnol. Biol. Med..

[95] Alidaee M.R., Taheri A., Mansoori P., Ghodsi S.Z. (2005). Silver nitrate cautery in aphthous stomatitis: A randomized controlled trial. Br. J. Dermatol..

[96] Ebrahimi S., Dabiri N., Jamshidnejad E., Sarkari B. (2007). Efficacy of 10% silver nitrate solution in the treatment of common warts: A placebo-controlled, randomized, clinical trial. Int. J. Dermatol..

[97] Niizeki K., Hashimoto K. (1999). Treatment of molluscum contagiosum with silver nitrate paste. Pediatric Dermatol..

[98] Cong T.X., Hao D., Wen X., Li X.H., He G., Jiang X. (2019). From pathogenesis of acne vulgaris to anti-acne agents. Arch. Dermatol. Res..

[99] Jurairattanaporn N., Chalermchai T., Ophaswongse S., Udompataikul M. (2017). Comparative trial of silver nanoparticle gel and 1% clindamycin gel when use in combination with 2.5% benzoyl peroxide in patients with moderate acne vulgaris. J. Med. Assoc. Thail..

[100] De la O-Escamilla N.O., Sidbury R. (2020). Atopic dermatitis: Update on pathogenesis and therapy. Pediatric Ann..

[101] Bekić S., Martinek V., Talapko J., Majnarić L., Mihaljević M.V., Škrlec I. (2020). Atopic Dermatitis and Comorbidity. Healthcare.

[102] Cookson W. (2004). The immunogenetics of asthma and eczema: A new focus on the epithelium. Nat. Rev. Immunol..

[103] Gauger A. (2006). Silver-Coated Textiles in the Therapy of Atopic Eczema. Biofunctional Textiles and the Skin.

[104] Benn T.M., Westerhoff P. (2008). Nanoparticle silver released into water from commercially available sock fabrics. Environ. Sci. Technol..

[105] Amin M., Azab M., Hanora A., Abdalla S. (2017). Antifungal activity of silver nanoparticles on Fluconazole resistant Dermatophytes identified by (GACA)4 and isolated from primary school children. Cell. Mol. Biol..

[106] Mousavi S.A.A., Salari S., Hadizadeh S. (2015). Evaluation of antifungal effect of silver nanoparticles against microsporum canis, trichophyton mentagrophytes and microsporum gypseum. Iran. J. Biotechnol..

[107] Hassan A., Mansour K., Mahmoud H. (2013). Biosynthesis of silver nanoparticles(Ag-Nps) (a model of metals) by Candida albicans and its antifungal activity on Some fungal pathogens (Trichophyton mentagrophytes and Candida albicans). N. Y. Sci. J..

[108] Noorbakhsh F., Rezaie S., Shahverdi A.R. Antifungal Effects of Silver Nanoparticle alone and with Combination of Antifungal Drug on Dermatophyte Pathogen Trichophyton Rubrum. Proceedings of the International Conference on Bioscience, Biochemistry and Bioinformatics.

[109] Drake P.L., Hazelwood K.J. (2005). Exposure-related health effects of silver and silver compounds: A review. Ann. Occup. Hyg..

[110] Tlili A., Jabiol J., Behra R., Gil-Allué C., Gessner M.O. (2017). Chronic Exposure Effects of Silver Nanoparticles on Stream Microbial Decomposer Communities and Ecosystem Functions. Environ. Sci. Technol..

